# On being (not so) different: perceptions of gender dysphoria and neurodiversity among people aged 15–35 in Sweden

**DOI:** 10.3389/fsoc.2025.1610206

**Published:** 2025-08-29

**Authors:** Fatih Özel, Gabriele Griffin

**Affiliations:** ^1^Department of Organismal Biology, Uppsala University, Uppsala, Sweden; ^2^Centre for Women's Mental Health During the Reproductive Lifespan – Womher, Uppsala University, Uppsala, Sweden; ^3^Centre for Gender Research, Uppsala University, Uppsala, Sweden; ^4^Center for Gender and Africa Studies, University of the Free State, Bloemfontein, South Africa

**Keywords:** gender diversity, gender dysphoria, neurodiversity, autism, Sweden

## Abstract

There has been an increase in the number of people experiencing gender dysphoria and neurodiversity over the last decade. Medical studies employing quantitative methodologies consistently report a high co-occurrence of gender diversity and neurodiversity. Simultaneously various sociocultural views have been proposed to understand these conditions together. Still, there is limited evidence on how this co-occurrence is experienced by individuals with gender dysphoria. This article aims to investigate how gender dysphoria and neurodivergent conditions, specifically autism, are articulated and perceived by people aged 15–35 experiencing gender dysphoria in Sweden. Sixteen semi-structured interviews, conducted between August 2023 and March 2024, were analyzed using thematic analysis following Braun and Clarke's approach. According to the themes identified in our data, the participants recognized themselves as divergent, with some suspecting that they might be neurodivergent. They were also familiar with the commonly reported co-occurrence of gender diversity and neurodiversity. Lastly, our participants discussed the intersectional relation between gender diversity and neurodiversity predominantly as a social rather than a clinical phenomenon, with neurodiversity in some instances seen as facilitating gender diversity. Our findings may reflect a newly emerging perspective on how the co-existence of gender diversity and neurodiversity is interpreted by those experiencing gender dysphoria.

## Introduction

Over the past two decades, the issues of gender, gender identity, and transgender have received significant attention in both academic and more general public spheres in many countries. One reason for the growing interest in gender identity, particularly in transgender and gender diverse identities, appears to be the increasing number of adolescents and young adults diagnosed with gender dysphoria, as indicated by various statistics (Goh et al., 2024; Jarvis et al., 2025; Kaltiala et al., 2020; Nyquist et al., 2025; Zhang et al., 2020). Along with the reported rise in gender dysphoria diagnoses and the increasing demand for medical interventions such as gender-affirming treatments, extended media coverage on the topic in diverse contexts has played a significant role in shaping perceptions of transgender and gender identity issues (Åkerlund, 2018; Indremo et al., 2022). Transgender and gender diverse identities, as well as gender dysphoria, have come under increased scrutiny, sometimes sparking controversy. These debates have included, but are not limited to, the provision of puberty blockers for minors (Rew et al., 2021), cases of transgender individuals who undergo a detransition for various reasons (MacKinnon et al., 2023), the more pronounced increase in gender dysphoria diagnoses among individuals assigned female at birth^1^ (AFAB) compared to those assigned male at birth (AMAB) (Indremo et al., 2021; Jarvis et al., 2025; Kaltiala et al., 2023), and the increasingly reported high co-occurrence of gender dysphoria and neurodivergent conditions (Goetz and Adams, 2024; Kallitsounaki and Williams, 2023; Özel et al., 2025). This study focuses on the last of these listed debates, aiming to explore how gender dysphoria and neurodivergent conditions—specifically autism, and to a lesser extent attention deficit hyperactivity disorder (ADHD)—are perceived and articulated by people with gender dysphoria. The article therefore takes a bottom-up approach to a topic that is often addressed simply from within medical contexts.

As the American Psychological Association (2015) defines it, gender identity is considered a person's deeply-felt—but potentially fluctuating—sense of being a girl, a woman, or female; a boy, a man, or male; or an alternative gender (e.g., genderqueer, gender non-conforming, gender-neutral). This sense of identity may align or differ from a person's sex assigned at birth, their physical traits, or how others perceive them. Diverse gender identities have been examined in the context of their intersections with other identity markers, including race, sexual orientation, and social class, among others (Robbins and McGowan, 2016). An increased focus on intersectionality has been called for when studying gender identities (Patterson and Cochran, 2021).

Being neurodivergent has also emerged as a form of identity, and the interconnections between gender identity and neurodiversity have been suggested to be addressed through the lens of intersectionality (Botha and Gillespie-Lynch, 2022). The existing literature on the co-occurrence of gender diversity and neurodiversity has predominantly employed a medical perspective, with a significant focus on the high co-occurrence of transgender and gender diverse identities and the potential reasons behind this overlap (Kallitsounaki and Williams, 2023; van der Miesen et al., 2016). Regardless of how this intersection of gender diversity and neurodiversity is approached or the reasons behind it, research on the perception of this intersection among individuals experiencing gender dysphoria remains scarce. Although several studies have addressed the experiences of people diagnosed with both gender dysphoria and autism (Coleman-Smith et al., 2020; Cooper et al., 2022, 2023; Oliver et al., 2025; Shimoyama and Endo, 2024; Strang et al., 2018), there is a lack of current research on the perception of neurodiversity and its relationship with gender dysphoria, not only in individuals with autism but on a broader gender dysphoria scale. This is particularly important for understanding how narratives about the intersection of gender diversity and neurodiversity are produced and perpetuated, including, importantly, among those experiencing gender dysphoria.

Before proceeding further, we would like to clarify the terminology used in this article; there is also more information with regard to some terms used in the sections below. Gender identity refers to the above-mentioned definition from the American Psychological Association. Transgender and gender diverse are not used interchangeably but together, to refer to individuals whose gender identities diverge from the social and cultural norms associated with their sex assigned at birth, regardless of whether they experience dysphoria about the misalignment between their gender identity and their sex as assigned at birth. Gender dysphoria refers to the discomfort caused by the mismatch between one's gender identity and one's sex as assigned at birth, but may or may not correspond to a clinical diagnosis, depending on the context. While we acknowledge the variety of conditions that may be referred to as neurodiversity in the literature (Armstrong, 2010; Botha and Gillespie-Lynch, 2022), we primarily focus on autism and, to a lesser extent, on ADHD as neurodivergent conditions in this article. The reasons for this are as follows: the neurodiversity concept largely emerged within autism communities, as described below; much of the existing neurodiversity literature is based on autism; and, most importantly, our study participants most often discussed autism and occasionally ADHD in relation to neurodiversity; and autism and ADHD were the only conditions exemplified during the interviews when informants provided examples. We use neurodiversity and neurodivergence interchangeably throughout this article. Lastly, there are differing perspectives on the preferred terminology for describing autism. Some individuals favor person-first language (e.g., “person with autism”), while others advocate identity-first language (e.g., “autistic person”) (Bury et al., 2023; Taboas et al., 2023). In this article, we use both forms to reflect the diverse preferences within autistic communities.

In the following sections, we outline the contemporary understanding of gender diversity, transgender identities, and gender dysphoria. We outline the concept of neurodiversity, including ongoing debates around the concept. This is followed by a brief overview of the literature highlighting the intersection of these two conditions: gender diversity and neurodiversity. We then present our results on perceptions of neurodiversity and its co-occurrence with gender dysphoria among individuals experiencing gender dysphoria in Sweden, and provide a discussion of our findings.

### Gender diversity

Gender diversity existed long before medical characterizations and classifications came into practice (Crocq, 2022). Issues related to sexual and gender identities, initially not clearly distinguished, entered the medical field through psychoanalytic practices. In early to mid-20th century Europe, homosexuality began to be differentiated from transsexualism (Drescher, 2014). Subsequently, Harry Benjamin's work (Benjamin, 1966) popularized the term “transsexual” and helped to establish it as distinct from “transvestite.” His contributions played a key role in shaping the endocrinological—and more broadly medical—understanding of transsexualism (Stewart, 2017). The American Psychiatric Association (1980) introduced the diagnostic category “transsexualism” for the first time in the third edition of their classification system for mental disorders, the Diagnostic and Statistical Manual (DSM). The same diagnostic category was called “gender identity disorder” in DSM-IV (American Psychiatric Association, 1994) and “gender dysphoria” in DSM-5, with some changes in the diagnostic criteria. Gender dysphoria is defined as “a marked incongruence between one's experienced/expressed gender and assigned gender” in DSM-5 (American Psychiatric Association, 2013). Gender incongruence is also a clinical diagnosis according to the 11th revision of the International Classification of Diseases (ICD-11) by the World Health Organization (2018). In ICD-11, the previous diagnostic category of gender identity disorder was replaced with gender incongruence, which is defined as “a marked and persistent incongruence between an individual's experienced gender and the assigned sex.” Gender incongruence is classified as a condition related to sexual health in ICD-11, in contrast to the previous categorization as a mental disorder. Even though these terms refer to clinical diagnoses, gender dysphoria—and sometimes gender incongruence—has been commonly used by transgender and gender diverse individuals, regardless of whether they have a clinical diagnosis (Davy and Toze, 2018). At the same time, it should be acknowledged that having a transgender or gender diverse identity does not necessarily lead to distress or gender dysphoria (Byne et al., 2018).

While gender diversity shifted from being considered illegal to being viewed as psychopathological, and later as a broader medical condition—particularly within medical disciplines—the conventional categories of gender, “women” and “men,” and the relationship between sex and gender became subjects of extensive discussion among feminist scholars. (Butler 1990) is one prominent scholar who challenged the prevailing assumption that sex is natural and gender is merely a social construct. Butler argued that both sex and gender are socially and culturally constructed, performed through repeated acts and practices. According to Butler, binary sex and gender constructs serve as regulatory structures that reinforce gender norms. From the 1990s onward, Butler's ideas, along with those of other contemporaneous scholars, contributed to the emergence of queer theory, a framework that critiques normative assumptions about gender and sexuality and emphasizes the transformative and intersectional nature of identities (Jagose, 1996).

The rise of the understanding of gender as socially constructed has also influenced psychological and medical approaches which have historically treated gender diversity as biologically and/or medically determined. This reframing has led to medical models that aimed to define and regulate gender diversity being increasingly criticized, particularly within the social sciences and humanities. These critiques, in part, influenced the aforementioned changes in diagnostic practices—despite ongoing criticisms of current frameworks (Ashley, 2021; Davy and Toze, 2018). At the same time, these frameworks continue to shape psychomedical understandings of gender diversity (Linander et al., 2021).

Following the drastic changes in the perception of gender diversity over the last century, the terminology used to describe different gender identities has become even more diverse in recent decades (Thorne et al., 2019). Contemporary views on gender have moved away from binary perspectives, and non-binary gender identities have increasingly been recognized among transgender and gender diverse individuals (Cheung et al., 2020; Scheim et al., 2021). Terms such as genderfluid, genderqueer, gender expansive, and agender, among others, have become part of the current lexicon (Ervin et al., 2023). These changes in gender terminology have taken place alongside broader shifts in public discourse, legislation, and medical interventions. Similar to views on gender diversity, understandings of neurodivergent conditions has undergone significant changes in recent years.

### Neurodiversity

Autism was initially described by (Kanner 1943) as an infantile condition characterized by problems in social interaction and resistance to change. Simultaneously, (Asperger 1944) defined a similar condition among young boys with social difficulties, unusual interests, and intact verbal skills. Together, these two physicians introduced the concept of autism, which was heavily debated regarding whether it represented a separate psychiatric phenotype until its first appearance in DSM-III. Autism was included in DSM-III as a new category of mental disorder under the name “pervasive developmental disorders.” The diagnostic criteria for autism were elaborated in DSM-IV, and Asperger's disorder was introduced as a separate but related entity. Based on the variability of autistic symptoms and the heterogeneity of autism manifestations among individuals, DSM-5 approached the phenomenon as a spectrum and used the term “autism spectrum disorder (ASD).” Similarly, ICD-11 embraced the same term in its latest version.

Criticism of these diagnostic criteria has been raised for various reasons, including methodological concerns and debates over the use of categorical vs. dimensional approaches, as well as the advantages and disadvantages of using a broader, single entity (Rosen et al., 2021). A systematic review of studies conducted from 2012 to 2021 across multiple countries indicated that the prevalence of ASD is about 100 cases per 10,000 individuals (Zeidan et al., 2022). Furthermore, a significant rise in the prevalence of ASD over the past few decades has been reported (Grosvenor et al., 2024). Factors such as changes in diagnostic criteria, heightened awareness of the condition, and improved access to healthcare services have been proposed as potential contributors to this increase in ASD prevalence (Hirota and King, 2023).

In contrast to the conventional medical classifications of autism, as outlined above, the neurodiversity concept emerged in the 1990s, primarily within online autism communities (Botha et al., 2024). Although the term's first use is often attributed to Harvey Blume and Susan Singer between 1997 and 1999, the initial ideas surrounding the concept can be traced back a few years earlier to an autistic community email list (Botha et al., 2024). Neurodiversity is defined in various ways by different scholars, but in its broader interpretation, it refers to acknowledging the diversity of minds, brains, and neurocognitive profiles among human beings, similar to the concept of biodiversity (Walker, 2014). It can also refer to a social movement that seeks to alter societal perceptions and responses to neurological diversity, or to specific perspectives on the neurocognitive differences that constitute this diversity (Hughes, 2021). There is no consensus on the precise definition of neurodiversity, or whether it should be considered as an approach, paradigm, movement, or a combination of them all in different contexts (Dwyer, 2022). However, the dynamic and evolving nature of the concept has been emphasized (Chapman, 2020).

There are certain specific controversies that have been extensively debated in the neurodiversity literature. Similar to the distinction between medical and social models of disability (see Oliver, 1990), there is an ongoing discussion about whether neurodivergent conditions should be understood entirely through a social or a medical model (Dwyer, 2022). For instance, (Singer 2016) has argued against both the strict medical model and the complete dismissal of biology by social approaches, while there have been different perspectives in the existing literature which either supported the social model or questioned its adequacy (Dwyer, 2022). One frequently raised concern is that the neurodiversity approach may not be applicable to individuals with severe intellectual disabilities or co-occurring medical conditions. It has been suggested that the concept of neurodiversity does not adequately capture the experiences of so-called “low-functioning” autistic individuals (Jaarsma and Welin, 2012). However, the categorization of individuals as “high-functioning” vs. “low-functioning” has itself been widely criticized (Flynn, 2018). The neurodiversity framework also highlights the strengths associated with neurodivergent brain structures and cognitive profiles (Baron-Cohen, 2017). Furthermore, the scope and boundaries of neurodiversity remain subjects of debate, with both proponents and critics including a broader range of neurological and psychiatric conditions under the term (Ne'eman and Pellicano, 2022).

Among the many discussions around neurodiversity, and autism in particular, the relationship with gender is especially noteworthy. Autism, originally conceptualized and diagnosed primarily in males, had long been framed as a male condition within medical disciplines. Clinical frameworks have historically been developed based on male cognitive profiles, which has led to the under-recognition of autistic presentations in females (Lonergan, 2021; Wood-Downie et al., 2021). This gender bias has attracted significant attention in recent years and has prompted investigations into how autistic traits in females may have been overlooked and how autistic traits may vary across genders (Cruz et al., 2025). There are also varied opinions on how to approach autism's intersection with gender diversity.

### Intersections of diversities

The intersection of gender diversity and neurodiversity is examined from various perspectives in the scientific literature, largely depending on the disciplinary context of the research. From a sociocultural standpoint, neuroqueer theory was introduced in 2014 by a group of activists and scholars, including Nick Walker (Barnett, 2024). Rooted in queer theory, neuroqueer theory centers on the intersection of two socially constructed norms—heteronormativity and neuronormativity—and offers critical perspectives on both (Walker, 2021). Within this framework, (Walker 2021) discusses concepts such as neuroqueering and neurodoing as opposed to *being* neurodiverse, critiques of neuroessentialism, and the influence of neuronormative structures on social expectations. Aligned with the principles of neuroqueer theory, neuroqueer feminism has emerged as an interdisciplinary field that integrates feminist theory into the analysis of neurodivergent experiences (Fox, 2025). Alongside growing academic interest in this intersection, online communities have played a significant role in shaping discussions about the intersection of gender diversity and neurodiversity, particularly by contributing to knowledge production around neuroqueer issues (Oswald et al., 2022). New terms such as neurogender and autigender have begun to surface to describe identities within gender diverse and neurodivergent communities (Barnett, 2024). Social media also appear to have a particular influence in this context, as they have increasingly become spaces where people express and explore neuroqueer identities (Oswald et al., 2022).

As neuroqueer theory began gaining momentum around 2014, so the co-occurrence of gender diversity and neurodiversity also started receiving increased attention in the medical field at approximately the same time. In 2010, de Vries et al. were the first to identify a heightened prevalence of ASD among children and adolescents referred to a gender identity clinic in the Netherlands, using a standardized diagnostic tool (de Vries et al., 2010). Following this initial finding, research on the co-occurrence of ASD and transgender and gender diverse identities has steadily grown (Rea et al., 2024). Elevated levels of autistic traits and/or higher rates of ASD diagnoses have since been observed among transgender and gender diverse individuals across different age groups and study contexts, employing a variety of autism assessment tools (Bouzy et al., 2023; Rea et al., 2024; Warrier et al., 2020). A recent meta-analysis estimated the pooled prevalence of ASD among individuals with gender dysphoria or gender incongruence to be 11%, which is significantly higher than its prevalence in the general population (Kallitsounaki and Williams, 2023). Although there has been considerable speculation regarding the underlying factors contributing to this high co-occurrence, conclusive evidence remains limited in the medical literature. Hypotheses include the involvement of various biological factors during brain development, as well as psychological and social explanations—for example, the suggestion that gender diversity may arise from difficulties in conforming to societal norms among autistic individuals (Wattel et al., 2024).

Our study centers on the reported perceptions of individuals who have experienced gender dysphoria. This bottom-up approach continues to be largely absent from the relevant literature. Below we detail our materials and methods.

## Materials and methods

### The participants and the research setting

A total of 16 individuals who self-identified as having gender dysphoria were recruited between August 2023 and March 2024. The eligibility criteria were being 15 years of age or older and having current or past experiences of gender dysphoria. The age criterion of 15 years was chosen because individuals at this age are considered to have sufficient experience to contribute meaningfully to the study questions, and 15-year olds in Sweden can contribute to this research without parental consent. The first 15-year old participant was recruited as the child of one of the authors' acquaintances, and the second was recruited through that participant.

The focus was deliberately placed on self-reported experiences of gender dysphoria rather than medically diagnosed cases in this study, as not all individuals experiencing gender dysphoria seek clinical evaluation or support. Due to the sensitivity of the topic and the potential challenges in reaching the target population, participants were recruited using a snowball sampling approach (Parker et al., 2019). Initial general recruitment was facilitated through announcements distributed via two internal university mailing lists. The first participant was recruited via these channels, and further participants either directly responded to the announcements or were referred by prior participants.

The first author conducted semi-structured, one-on-one interviews with the participants, twelve in person and four via Zoom. Prior to the interviews, basic sociodemographic data were collected. All interviews were conducted in English, which was a second language for both the first author and 13 participants. The primary reason for conducting the interviews in English was that the researchers involved in the project were not native Swedish speakers. Given that 12 out of 16 participants were also not native Swedish speakers, we think that English served as a reasonable common language. This was also advantageous in the sense that the relevant academic literature is predominantly in English, and much of the evolving terminology related to trans identities originates from and continues to develop in English. One drawback, of course, may be that the use of English limited participants' ability to express themselves in the most nuanced manner. But we would have found it difficult to cover all the languages they represented.

The interview guide addressed issues including childhood and adolescent experiences related to gender, gender self-identification, gender expression, coexisting mental health conditions, views on biomedical research, and future expectations with regard to gender. Participation in our study was entirely voluntary and uncompensated. Participants were informed that they had the right not to answer any questions they found uncomfortable, to withdraw from the study at any stage, and that only pseudonymized data would be used in any dissemination. The interviews lasted between 48 and 90 min (mean duration: 64 min).

The average participant age was 26.7 years, ranging from 15 to 35. Ten were AFAB, and six were AMAB. Most participants were highly educated; nine held either a Master's or doctoral degree, which may be an effect of the recruitment strategy. While all participants were living in Sweden at the time of the study, their countries of origin varied and included several European countries, the United States, Russia, and India. Only four participants were Swedish, which may be related to the fact that the researchers themselves are not originally from Sweden. A brief description of the participants' demographic characteristics is provided in Table 1.

**Table 1:** Demographic information of study participants at the time of the interview.

Level of education	2 Ph.D. degrees; 7 Master's degrees;
	2 Bachelor's degrees;
	3 high school degree; 2 in 8th grade
Current employment	5 Ph.D. students; 2 Master's students; 2 Bachelor's students; 2 postdoctoral researchers; 2 secondary school students;
	1 data steward; 1 video game designer; 1 teacher
Living status	3 married and living with their partners;
	2 aged under 18 years and living with their parents; 11 legally single and living with/without their partners

This study received ethical approval from the Swedish Ethical Review Board (Dnr: 2023-01260-01).

### Analytical approach

The first author transcribed all the interviews verbatim and, together with the second author, read them multiple times to become deeply familiar with the material. The authors initially read the transcripts independently, then discussed their respective interpretations. The first author conducted the initial round of coding, after which both authors collaboratively refined the codes through iterative discussions. This process was guided by Braun and Clarke's (2022) reflexive thematic analysis. We would describe the coding process as both inductive, with themes directly identified from the data, *and* deductive, informed by the conceptual framing of the study. The interview data included themes that reflect how gender dysphoria, neurodiversity, and the relationship between those two are perceived by transgender and gender diverse individuals who experience gender dysphoria.

The analytical process resulted in the identification of four distinct themes that we address in this article: (1) recognition of being divergent, (2) suspicion of being neurodivergent, (3) awareness of the co-occurrence of gender diversity and neurodiversity and (4) neurodiversity as a challenge to normative expectations.

In writing up the data, we took particular care to avoid including any identifying details in the presentation of the findings, especially in direct quotes. We were also mindful of the potential for misrepresentation or overgeneralization. Therefore, we contextualized quotes carefully, providing enough background to preserve meaning without compromising anonymity.

## Results

The majority of our participants (10 out of 16) identified their gender identity as non-binary, either alone or in combination with other terms (Table 2). During the interviews participants used multiple terms to describe their gender identities, and it became clear that these terms had changed over the course of their lives. Perceptions of neurodiversity, and how participants positioned their (gender) identities in relation to it, were generally consistent, with some expected variations.

**Table 2:** Gender identifications of study participants at the time of the interview.

**Pseudonyms**	**Age (years)**	**Gender identificatory term**
Arina	34	Genderless
Asher	31	Non-binary, agender
Carmen	24	Non-binary
Carol	30	Non-binary
Celeste	31	Genderfluid
Harper	26	Non-binary woman
June	20	Genderless
Kai	29	Trans non-binary
Luca	24	Agender
Margo	30	Non-binary, trans masculine
Matthew	15	Demiboy
Mia	15	Non-binary
Quinn	30	Non-binary
Risha	22	Non-binary
Rowan	31	Non-binary
Victoria	35	Binary trans woman

### Recognition of being divergent

The participants in this study often spoke about being perceived as different by others—sometimes when recalling childhood memories, and at other times when describing more recent life experiences. It was quite common for them to refer to themselves as “weird” when talking about feeling like outcasts in their social environments. All participants expressed a sense of recognizing themselves as divergent, for varied reasons. Some examples of this recognition are:

I think that's reflected throughout my all, all of my education by ending up always in groups of people that were similarly outcasts …. this progressed throughout my education, the more, we kind of narrowed down in the funnel. So, we ended up being the weird kids in STEM [referring to science, technology, engineering, and mathematics] together. (Carol, non-binary, 30)

People know me in school as the gay kid, because they don't even really know the word trans or non-binary. They just know me as, oh, that's the weird gay kid. (Mia, non-binary, 15)

I've always felt out of place. Like I've always felt, like I'm the weird one, and everything, and I don't know, it's just a feeling I have. (Matthew, demiboy, 15)

I've never been like included in the village where I grew up. I was always the, the outcast, the freak, the gay. (June, genderless, 20)

Some participants emphasized how being perceived as divergent by others affected their social relationships, particularly during childhood and early adolescence. The acceptance they experienced from others varied greatly across participants and, for some, even depending on the context. While some faced more challenging experiences, others had supportive people around them. Overall, all participants encountered some degree of difficulty during their coming-out process. They described how they were perceived by others as follows:

I was a little freak …. I didn't fit in, but I didn't fit in with anything. Like this is, this is probably one of the reasons I was so far outside of the, I couldn't really connect with anyone. (Asher, non-binary/agender, 31)

I didn't really have a lot of friends. So, I was, because I was so like, weird for most people, and there were not, no, no, there were no friends. (Kai, trans non-binary, 29)

While Asher above reflected on being perceived as divergent due to gender-related matters, some other participants associated their sense of divergence with the fact or possibility that they were autistic:

I always kind of struggled making friends. Just because I didn't find this out until, way, way, way late, and too as an adult then, I'm also on the autism spectrum. And so, it was, it's a little strange making friends sometimes. I tended to make friends with other weird kids. (Harper, non-binary woman, 26)

[talking about difficulties they experienced during childhood] I must have been just, just awkward and weird already, then. I guess autism makes me a bit weird. (Quinn, non-binary, 30)

And I've started to accept that my brain is just wired a bit differently. That's a process in and of itself. But not, not knowing that, not knowing that when I grew up, meant that I just felt like I was always different. I could not relate to other people at all, it was so hard. (June, genderless, 20)

### Suspicion of being neurodivergent

At the time of the interviews, only one participant had an official autism diagnosis, received during their childhood. Six participants spoke about their suspicions of being autistic, with one of them actively seeking healthcare for a potential diagnosis. The reasons behind their self-suspicions of neurodivergence varied and were often not obvious:

I am not officially diagnosed to be on the spectrum. But my friends that are, that have shared all of their diagnostic tests. I ended up being very, very, very highly on them for, for autism. I don't know if my failure to fit in a group, or feeling like always didn't belong there. If it's part of, you know, being neurodivergent, or part of being very fluid in my gender identity. (Carol, non-binary, 30)

I probably have like, some tendency toward autism, like, everyone I've ever spoken to is, like, are you sure you don't have Asperger's, which we don't call it anymore, but you know. (Victoria, binary trans woman, 35)

This first person that I, that I met, that was non-binary. They were autistic. And, and I want to do the test because I feel like I have something. (Carmen, non-binary, 24)

I think I'm quite autistic. And actually, my child is officially diagnosed with autism … [in response to if she had a diagnosis] I'm not diagnosed with autism. (Arina, genderless, 34)

Regarding ADHD, one participant mentioned having an official diagnosis. Another participant shared that they had suspected having ADHD during childhood, while two others spoke about their current suspicions at the time of the interviews—both of whom were actively seeking healthcare.

I don't have an official autism diagnosis, I have an official ADHD diagnosis. But I strongly suspect that I have autism and doing self-therapy for autism. (Harper, non-binary woman, 26)

### Awareness of the co-occurrence of gender diversity and neurodiversity

In line with the increased attention to the co-existence of gender diversity and neurodiversity in recent years—and the debates surrounding the topic—all participants except one were familiar with the high co-occurrence of these two phenomena. The extent of familiarity varied among the participants, likely in connection with their own suspicions of being neurodivergent. Many had also encountered the topic through online sources:

I think I heard it about like four or five years ago, maybe, which, because I know that, when people like go online, people, instead of using the term disability for autism, or ADHD, they just call it neurodiversity. Okay, it's a new term. And sometimes they call atypical and typical. (Risha, non-binary, 22)

… from the stuff that I have been reading in the last 10 years and research in this field, there is a huge overlap between these two things. That is neurodivergent people have the highest rate of transgender and non-binary identities. (Carol, non-binary, 30)

[referring to issues on neurodiversity] I have seen some of them, but I haven't, I don't, I used to engage a lot. We've kind of like discussions, like that, online. (Mia, non-binary, 15)

Some participants also shared experiences of knowing gender diverse and neurodivergent individuals in their social circles, which contributed to their understanding of the intersection between gender diversity and neurodiversity:

I'm not officially diagnosed with anything like neurodivergent. But I definitely have seen like the connection, or like, the prevalence of like gender diversity in your neurodivergent groups and people, and I just think it's very interesting. I don't know, I don't really have any, you know, ideas about what that might be, but I've definitely seen the connection, or like the, yeah, that those two groups like overlap a lot … [describing their first encounter with the neurodiversity concept] at first, mainly online, when I wasn't, when I didn't, like how many, I don't know, like when I didn't have a lot of diverse people in my friend group. But definitely now, in person, I had experiences with that as well. And yeah, I think, like all, like almost all trans people I know slash I've met in the past couple of years have been neurodivergent. (Luca, agender, 24)

A lot of my friends are trans and a lot of my friends are neurodiverse. (Mia, non-binary, 15)

I saw quite a lot of research. I think people, trans people who I communicate with, there are quite a lot of autistic people. (Arina, genderless, 34)

### Neurodiversity as a challenge to normative expectations

Following the discussion about participants' feelings of being divergent, their positioning in relation to neurodiversity, and their familiarity with the co-occurrence of gender diversity and neurodiversity, the interviews also explored their perceptions of this intersection and how they interpreted it. All participants who were familiar with the concept described the co-existence of gender diversity and neurodiversity as a reflection of neurodivergent individuals' rejection of various societal norms, including the gender-related norms. In other words, autistic people were seen as less likely to conform to gender norms, and therefore more likely to express gender diversity. This was typically expressed as follows:

There is a huge overlap between neurodivergent people and people that express their gender differently, or feel their gender differently. Because they don't, they don't see the need to conform with this societal norm or with the societal idea of what your gender should be … Neurodivergent people struggle to accept man-made social norms that just feel very arbitrary and not natural, and social construct of gender is one of them. (Carol, non-binary, 30)

[referring to neurodivergent people] They're not like, they don't feel like they have to be boxed in by stereotypes, because they're already like, kind of atypical … they might just, like, want to explore a bit more, I guess. (Matthew, demiboy, 15)

I think that autistic people more often question themselves, so their lives, they reflect on some norms imposed on them. So, that's why they can be more likely to identify as trans. (Arina, genderless, 34)

[referring to neurodivergent people] They ask themselves questions actually. And they, they rethink about the system. And of course, like, they don't match with the system. (Carmen, non-binary, 24)

I would say both autism and gender, being non, non-cis, both of these are statuses, conditions where you are more aware of what society expects of you. And these sort of made up implicit rules that society has, and expectations of how you behave and present yourself, and in both cases, you are more willing to challenge those rules. So, for me, I think it kind of makes sense, this, this, this correlation between being trans and being neurospicy [last word as used by informant]. (Asher, non-binary/agender, 31)

One participant talked about how they observed the relationship between being neurodivergent and gender norms in their sibling as follows:

Many people with neuro, neurodivergence tend to, you know, explore their gender more, or find like a stable point or something. Because, for example, my sibling, he, he's a, again, he doesn't know what is feminine, what is masculine … and that may be, might be associated with the factor that your gender isn't really something that's fixed when you are neurodivergent. (Risha, non-binary, 22)

According to some participants, in line with the difficulties autistic individuals face in adhering to normative expectations, being neurodivergent can facilitate the recognition of gender diversity and, in some cases, feelings of gender dysphoria:

A lot of neurodiverse people don't really see those, those boxes as much, or don't feel inside of those boxes anyways. That, it might be more, that it's easier for them to recognize gender dysphoria, as recognize and come out as such, rather than actually the incidents being higher in neurodiverse people. (Kai, trans non-binary, 29)

People who are neurodivergent are more likely to, to recognize, you know, queerness itself and in others because, because of that, and I think because they have less like judgment attached to it. Because they're less invested in, in the story of gender that we're taught, told. (Rowan, non-binary, 31)

That autistic people are bad at adapting to expectations, and therefore, they're also bad at adapting to gender expectations. And therefore they, they, they show, they're closer to the true occurrence of dysphoria in the population, because they're unable to mask it the way that, that non-autistic people would do. So that's sort of my favorite hypothesis. (Victoria, binary trans woman, 35)

I don't think that there's a higher rate of people who are neurodivergent and trans … I think there's a higher rate of neurodivergent people who realize that they're trans or non-binary, vs. neurotypical people who realize that they're trans non-binary. (Harper, non-binary woman, 26)

Unlike other participants, Mia also emphasized the way both gender diverse and neurodivergent individuals are perceived by others in relation to the intersection of gender diversity and neurodiversity:

If you are neurodiverse, and you're often told that you're different, or you're weird, or whatever. Then it's similar if you're trans, so I guess there's communities that kind of, like, have a similar thing in sort of, like a similar, similar experience, and I suppose communities like that, often kind of, you know, like, are connected. (Mia, non-binary, 15)

## Discussion

In this study, we aimed to investigate perceptions of neurodiversity and its intersection with gender diversity among people experiencing gender dysphoria in Sweden. Our results revealed that participants, aged between 15 and 35 and using diverse gender identificatory terms to describe their identities, shared a common experience of feeling different and/or divergent across various contexts. Almost all participants were also familiar with the intersection of transgender and gender diverse identities and neurodivergent conditions. It was relatively common for participants to suspect they might be autistic or to embrace a self-diagnosis of autism. Online sources were the primary means through which participants learned about this intersection. Regarding the intersection of two phenomena in focus in our study, the most common interpretation articulated by the participants was that autism might free individuals from conforming to gender norms, thereby creating space for gender diverse identifications. Variations of this main interpretation were expressed by a number of the study participants.

Among the participants in this study, it was particularly salient that both gender diversity and neurodiversity were predominantly conceptualized as social rather than medical phenomena. Moreover, within the structures of these social phenomena and how they operate, peer culture played a significant role. This peer culture could be exclusionary, as reflected in all participants articulating feelings of being different and of somehow being outliers in their social milieu. This sense of divergence may have shaped their exploration of gender identity, as further elaborated by some participants. Notably, many participants described being perceived as divergent by others, rather than inherently seeing themselves as different. The importance of social attribution was especially evident in how it shaped participants' self-perceptions. At the same time, peer culture in connection to gender diversity and neurodiversity could also be inclusionary, as illustrated by several participants. Being labeled as divergent in childhood sometimes translated into belonging to a (neurodivergent) community later in life—for instance, described by one participant as being “weird kids in STEM.” Overall, it was clear that peer culture and others' reactions played a significant role in how participants positioned themselves in relation to the notion of divergence.

In terms of neurodivergence, a few participants explicitly connected their feelings of being different to their (self-diagnosed) autism. The concept of “feeling different” was also evident in a study by (Coleman-Smith et al. 2020) in the UK, where ten adults diagnosed with both gender dysphoria and autism were interviewed. Their findings suggested that the intersection of diverse experiences related to gender and autism contributed to this feeling of difference, with autism in some cases amplifying it. Similar findings were reported by (Cooper et al. 2022), who conducted a qualitative study with 21 transgender and/or non-binary adults with autism in the UK. Participants in that study also described challenges associated with the experience of feeling different. Although the implications of this commonly reported sense of divergence are not entirely clear from these studies, including our own, it appears to be an important angle to explore in order to deepen our understanding of the early life experiences of transgender and gender diverse individuals, both with and without autism.

Attention to the intersection of gender diversity and neurodiversity has increased over the past two decades, both in academic and lay communities, as outlined in detail in the introduction. In line with this trend, all but one of our participants were to varying degrees familiar with ongoing discussions about gender diversity and neurodiversity, particularly through online platforms. Among the participants in this study, the relationship between gender diversity and neurodiversity was described in a way that could be viewed as both normative and normalized. All participants interpreted the intersection through a social lens, often framing autism as preceding gender diversity and facilitating gender diverse identities and expressions. They also shared examples from their social environments that supported this interpretation. Similar conceptualizations of the intersection between gender diversity and neurodiversity have been reported in previous qualitative studies involving autistic individuals experiencing gender dysphoria (Cooper et al., 2022, 2023). In our study, no problematic or negative aspect of framing a normative relation between neurodiversity and gender diversity was raised by the participants. On the contrary, the imbrication of gender diversity and neurodiversity was presented as a naturalized situation, something of an emerging dominant, normative narrative.

The number of participants in this study who suspected being neurodivergent, mostly autistic, or who self-diagnosed as autistic, was particularly noteworthy. Indeed, the phenomenon of autism self-diagnosis has been examined by several researchers over the past decade, beginning with the initial investigations by (Kapp et al. 2013). An increase in autism self-diagnoses and the role of online communities and forums in this increase has been suggested (Lewis, 2016, 2017). According to a review by (Overton et al. 2024), which evaluated studies on autism self-diagnosis conducted between 2000 and 2021, several factors have been associated with this phenomenon. These include differing views about medical care and related experiences, different understandings of autism, and whether autism is perceived as an identity marker or not. While the motivations or reasoning behind participants' suspicions of being neurodivergent were not clearly articulated in our interviews, the issue of autism self-diagnosis was evident.

Another particular aspect in the perception of gender diversity and neurodiversity among our participants was that one kind of diversity seemed to create openness to another kind of diversity—for instance, being neurodivergent appeared for some to facilitate experimenting with gender norms and created a willingness to embrace gender diversity. While this view may indicate a newly emerging perception of the intersection of these two diverse conditions, it should be acknowledged that this view was prominent in a group where individuals' gender diverse features were more pronounced than their neurodiverse features. Neurodivergent individuals might have different views on whether being diverse in a particular context facilitates other diverse identity markers. Indeed, certain experiences of neurodivergent people reported in the literature contradict this view, where neurodiversity is framed as a challenge to negotiating gender diversity, due to particularities of the thinking processes among neurodivergent people (Coleman-Smith et al., 2020; Cooper et al., 2023). Taken together, gender diversity and neurodiversity may not have the same impact on attitudes toward other forms of diversities and may play different roles in how individuals approach other diverse identifications. In other words, the primary diversity category with which an individual identifies may determine how this individual relates to other identity markers.

## Conclusion

In this study, we explored perceptions of neurodiversity and its intersection with gender diversity among individuals experiencing gender dysphoria. A high co-occurrence of gender diversity and neurodiversity across different contexts was recognized by the study participants. Both gender diversity and neurodiversity, as well as their intersection, were viewed as social constructs, and emerge as a new dominant narrative among this group. Our results suggest that this indicates at least to some that these two forms of diversity merge at a socio-discursive level. At the same time, there is accumulating medical evidence demonstrating this intersection through a high rate of co-occurrence. Given the range of findings on this issue, the meaning of the intersection of gender diversity and neurodiversity for individuals' sense of themselves warrants further research.

## Data Availability

The datasets presented in this article are not readily available because the data used in this study cannot be shared due to privacy requirements. Requests to access the datasets should be directed to fatih.ozel@ebc.uu.se.

## Footnotes

^1^We use the phrase “sex assigned at birth” which is common in much contemporary literature on this topic but would like to note that “sex one is assumed to have” is another formulation also in use.
